# The Influence of Competitive Level on Sleep Quality and Quality of Life in Adolescent Soccer Players

**DOI:** 10.7759/cureus.71395

**Published:** 2024-10-13

**Authors:** Mourad Ahmedi, Sebastian Sitko, Carmen Mayolas-Pi

**Affiliations:** 1 Physiatry and Nursery, University of Zaragoza, Huesca, ESP

**Keywords:** adolescent soccer players, competition level, psychology, quality of life, sleep quality

## Abstract

Introduction

Soccer is one of the most popular sports worldwide and is a leading physical activity choice among adolescents. This study aims to investigate how the competitive level of adolescent soccer players impacts their sleep quality and quality of life.

Methods

A total of 5,692 adolescents aged 11 to 19 years were classified based on their physical activity levels and competitive engagement. Participants were categorized as inactive, non-competitive athletes, or soccer players competing at local, regional, or national levels. An anonymous online questionnaire was used to assess sociodemographic characteristics, physical activity level, competition level, sleep quality, and quality of life. Significance was set at p < 0.05. The Chi-square (χ²) test was employed to examine frequency distributions. Comparisons based on competitive level were performed using Generalized Linear Models, adjusting for age, BMI, municipality size, and economic level. When significant differences were identified, post hoc analyses were conducted with a Bonferroni adjustment.

Results

Girls reported lower sleep quality and quality of life compared to boys. Soccer players exhibited better sleep quality and life satisfaction than inactive individuals and non-competitive athletes, independent of competitive level (p > 0.05 Bonferroni pairwise comparison). Among boys, regional-level soccer players showed the highest sleep quality and quality of life. For girls, national-level soccer players reported the best outcomes in both parameters.

Conclusion

Adolescent soccer players, regardless of their competitive level, demonstrate better sleep quality and quality of life than inactive individuals or non-competitive athletes. In general, higher levels of competition correlate with improved sleep quality and quality of life, particularly among female athletes.

## Introduction

Engaging in regular physical activity, including participation in sports, offers numerous health benefits for adolescents, positively impacting their mental, emotional, and physical well-being. A recent extensive analysis of the relationship between physical activity and psychological health demonstrated that sports serve as a valuable tool for emotional regulation, mood enhancement, and the development of social skills [1]. Increased participation in team sports during school years is also associated with reduced stress and loneliness, as well as improved sleep satisfaction in adolescents [2]. Finally, physical activity benefits several domains of cognition and metacognition in youth, including non-executive cognitive functions, core executive functions (working memory, selective attention inhibition, cognitive flexibility), and metacognition [3].

Adolescence is a critical developmental stage marked by profound behavioral, social, and emotional transformations. It plays a pivotal role in shaping mental well-being and social habits. According to the World Health Organization, mental health problems emerging during adolescence often persist into adulthood, emphasizing the importance of early interventions to safeguard future mental health [4]. Adolescence is also characterized by significant physiological, psychiatric, sociocultural, and psychological changes, all of which influence health outcomes, including sleep quality and quality of life (QoL) [5]. Further, adequate sleep is essential for optimal physical, cognitive, and emotional development during this period [6]. While research on sleep often focuses on duration, sleep quality has shown even greater implications for health, with individual variations [7]. Adolescents commonly experience a decline in both sleep duration and quality, alongside an increase in sleep disturbances, particularly among females [8]. A recent systematic review demonstrated that regular physical activity reduces sleep latency, enhances overall sleep quality, and aids in the management of sleep disorders such as insomnia [9]. Moreover, evidence suggests that adolescents who engage in physical activity exhibit better sleep quality than their inactive peers, with higher activity levels correlating with improved sleep outcomes [10]. Finally, high levels of physical activity in adolescents are associated with distinct neurophysiological patterns, including specific electroencephalography (EEG) frequency characteristics and enhanced brain connectivity, as observed in both EEG and MEG studies [11]. These patterns are linked to better cognitive function, mental health, and behavioral regulation, highlighting the positive impact of physical activity on adolescent brain development.

Quality of life (QoL) is intimately tied to developmental stages and refers to individuals' perceptions of their position in life, influenced by their goals, expectations, values, and cultural context [12]. Adolescent quality of life is particularly multifaceted, influenced by gender, age, family dynamics, socioeconomic status, self-perception, health behaviors, and community environment. Research indicates that individuals who regularly participate in physical activities, particularly competitive sports, report higher life satisfaction than those who do not engage in such activities. As youth participation in sports continues to grow, understanding the relationship between sports competition and QoL is essential for promoting health and lifelong physical activity [13]. Adolescents involved in high-intensity physical activity tend to exhibit higher levels of school satisfaction compared to those engaging in low-intensity activities, contributing to their emotional development [14]. Moreover, regular participation in sports is linked to greater body appreciation, which in turn leads to enhanced life satisfaction, as well as improved social and emotional health [15]. Adolescents engaging in high levels of physical activity report better QoL and higher self-efficacy, with more than 30 minutes of physical activity on five or more days per week being strongly associated with higher QoL [16]. Overall, physical activity improves QoL and psychological well-being, with sports participation contributing to greater life satisfaction, emotional health, and self-efficacy.

Globally, soccer stands out as the most popular sport, and it is among the top choices for adolescents [17]. Soccer is a dynamic, intermittent sport that requires both strength and power, characterized by alternating periods of high-intensity activity and sub-maximal effort over approximately 90 minutes. Players cover distances of 9-12 kilometers during a match, with the intensity ranging from 70% to 75% of their Maximal Oxygen Uptake (VO2 max), depending on position and play intensity [18]. Participation in soccer, like other sports, is an effective way for adolescents to enhance their physical activity and fitness levels, while also fostering enjoyment [19]. Soccer has also been shown to improve sleep quality and QoL, including aspects such as autonomy and social interaction [20]. However, the influence of competitive level on these outcomes remains unclear.

Despite the wealth of research highlighting the benefits of physical activity for adolescent health, there is a notable lack of studies examining the effect of sports competition level on sleep quality and QoL in this population. To the best of our knowledge, no study has yet explored the impact of competition level on sleep quality and QoL in adolescent soccer players. This study aims to fill this gap by examining how competitive level in soccer influences sleep quality and QoL in adolescent players. Specifically, we seek to evaluate the influence of competition level on these key health parameters.

## Materials and methods

Design

This cross-sectional study is based on self-reported data, conducted in accordance with the Declaration of Helsinki, and was approved on 18/10/2017 by the Clinical Research Ethics Committee of Aragón (PI/0339). Adolescents aged 11 to 19 years were selected from three distinct population groups: (1) inactive individuals, (2) individuals who engage in sports without competing, and (3) individuals who play soccer and compete at various levels (local, regional, and national). For the inactive and recreational sports groups, secondary education institutions and vocational training centers across representative regions of Spain were contacted. For the soccer-playing participants, grassroots and national-category sports clubs were targeted. A comprehensive list of clubs representing both categories was compiled.

An initial email containing detailed information about the study, followed by a postal letter, was sent to each club. Subsequently, a follow-up phone call was made to establish contact with the person in charge of each club. For schools and clubs that agreed to participate, a link to the online questionnaire was provided. The questionnaire was entirely anonymous, and those responsible for distribution sent the link to the adolescents. By completing the questionnaire, the participants gave their consent for their data to be used in the research. The questionnaire, which was composed of the points highlighted below, remained open for a limited period, from January to March, around mid-season, to minimize any influence from periods of heightened competition.

Participants

All participants were recruited based on the general inclusion criterion of being free from any chronic diseases. Inactive subjects had to show a low level of physical activity according to the Physical Activity Questionnaire for Adolescents (PAQ-A) criteria and the Program of All-Inclusive Care for the Elderly (PACE) criteria. Non-competitive athletes had to state that they did any sports at least twice a week but did not compete in any sport. Soccer players had to train at least two days per week, with a minimum of six months of experience in the sport. All adolescents who could not be classified into one of these three groups were excluded. The total sample consisted of 5,692 adolescents, including 3,001 boys (mean age: 15.0 ± 1.7 years; mean BMI: 20.7 ± 3.7) and 2,691 girls (mean age: 15.0 ± 1.7 years; mean BMI: 20.4 ± 3.3). Participants were categorized into three groups: a non-athlete inactive control group (n=1,031), athletes who do not compete but have trained for at least two days per week over the past six months (n=3,249), and soccer players (n=1,412). Among the soccer players, 1,080 competed at the local level, 165 at the regional level, and 167 at the national level. 

Outcomes

The initial questions focused on the participants' sociodemographic characteristics, socioeconomic status, and level of competition. Subsequently, a series of validated questionnaires were administered to assess key outcomes:

Physical Activity Level, Training, and Athletic Performance

A custom questionnaire was designed to evaluate several aspects of soccer-related activity, including volume (hours per week), frequency (days per week), training experience (years of soccer training), and performance level (local, regional, or national competitions).

The PAQ-A, a validated tool with robust psychometric properties, was used in its Spanish version [1,21]. This questionnaire assesses physical activity over the past seven days, including activities during free time, physical education classes, and other schedules. The PAQ-A consists of nine questions that assess various dimensions of physical activity on a 5-point Likert scale, although only eight questions contribute to the final score. The final score was derived by calculating the arithmetic mean of the scores from these eight questions.

Additionally, a specific question was included to assess physical activity over the past two weeks [22]: “During the past two weeks, on how many days were you physically active for a total of at least 60 minutes? For each day, add up all the time you spent in physical activity, like walking, riding a bicycle, etc. Do not include physical education or gym class. Count up the days with at least 60 minutes of physical activity in the past two weeks.” Responses ranged from 0 to 14 days.

To be included in the control group or classified as non-athletes with low physical activity (PA) levels, adolescents had to meet three conditions regarding physical activity/sport: 1) Not participating in competitive sports, 2) Scoring ≤2.9 on the PAQ-A, and 3) Engaging in 0 to two days per week of at least 60 minutes of moderate to vigorous physical activity, as per the PACE criteria. The PAQ-A cutoff criterion was used only to exclude inactive subjects who, while meeting two of the inclusion criteria, exhibited a physical activity level that, according to Voss et al., meets physical activity guidelines. This PA level data was not used as a cutoff point for the other groups [22].

Sleep Quality

Sleep quality over the past month was assessed using the Pittsburgh Sleep Quality Index (PSQI) [23]. This questionnaire evaluates seven components of sleep quality: (1) sleep duration, (2) sleep disturbances, (3) sleep latency, (4) daytime dysfunction due to drowsiness, (5) sleep efficiency, (6) overall sleep quality, and (7) use of sleep medications. Each component is scored on a scale from 0 to 3, with the total score ranging from 0 to 21. Higher scores reflect poorer sleep quality. For the purpose of analysis, the PSQI score was dichotomized as either good (PSQI ≤ 5) or poor (PSQI > 5) based on a previously established cut-off [23].

Quality of Life

The Health-Related Quality of Life Questionnaire for Children and Adolescents (KIDSCREEN-52), in its Spanish version, was used to assess the quality of life [24]. This questionnaire contains 52 items that evaluate ten dimensions: physical well-being, psychological well-being, mood, self-perception, autonomy, relationships with educators and family life, friends and social support, school environment, social acceptance, and economic resources. A 5-point Likert scale was employed to assess frequency or intensity. The value for each dimension was calculated as the sum of the responses to a specific set of items. From these sums, Rasch scores were derived for each dimension, with higher scores indicating better quality of life (QoL).

Sociodemographics

In addition to collecting data on age and BMI, sociodemographic information was gathered using two questions related to economic status: (1) "What do you perceive as the economic level of your family?" (with response options: Very Low, Low, Medium, High, or Very High) and (2) "Do your parents have trouble making ends meet?" (with response options: Yes/No). These questions are derived from the Family Affluence (FAS) scale [25].

Statistical Analysis

For statistical analysis, we utilized the IBM Statistical Package for the Social Sciences (IBM SPSS Statistics for Windows, version 20.0; IBM Corp, Armonk, NY, USA), with a significance level set at p < 0.05. Descriptive statistics, including means and standard deviations, were calculated for the variables assessed across the different study groups. Percentage analyses were conducted for quantitative variables. The Chi-square (χ²) test was employed to examine frequency distributions. Comparisons based on competitive level were performed using Generalized Linear Models, adjusting for age, BMI, municipality size, and economic level. When significant differences were identified, post hoc analyses were conducted with a Bonferroni adjustment.

## Results

Table 1 shows that the male and female samples are of similar age. However, girls are generally less physically active, train less, and report poorer sleep quality and quality of life compared to boys, with the exception of social domains. The distribution of municipality size and family economic level also differs significantly between genders (both, χ²=0.000). Since these characteristics may influence sleep and quality of life, they are considered as confounding variables in our analysis.

**Table 1:** Sample characteristics and comparison according to sex Values ​​expressed as mean (standard deviation) or percentage (%). ^1^BMI = Body Mass Index; ^2^inh = inhabitants. For quantitative variables, the General Linear Model adjusted for age, BMI, municipality size, and economic level was used: * p < 0.05 vs. boys. For qualitative variables, differences were examined using the Chi-square test: ** χ² < 0.05.

	Total (n = 5692)	Boys (n = 3001)	Girls (n = 2691)
Age	15.0 (1.7)	15.0 (1.7)	15.0 (1.7)
BMI^1 ^(kg/m2)	20.6 (3.5)	20.7 (3.7)	20.4 (3.3)*
Economic level (%)			
Very Low	0.5	0.4	0.6
Low	6.0	5.6	6.6
Medium	72.1	70.2	74.2
High or Very High	21.4	23.8	18.6
Trouble economics (%)			
Yes	9.9	8.7	11.1**
Residence size (%)			
≤1,000 inh^2^	4.9	4.3	5.6
1,001-10,000 inh	18.2	16.3	20.2
10,001-100,000 inh	28.7	28.2	29.3
100,001-500,000 inh	11.3	11.0	11.5
≥500,000 inh	37.0	40.2	33.3
Physical activity/training			
Years of practice	5.1 (3.4)	6.0 (3.7)	3.8 (3.1)*
Hours/week of practice	4.3 (3.0)	5.1 (3.3)	3.3 (2.1)*
Days/week of practice	3.2 (1.5)	3.5 (1.4)	2.8 (1.5)*
Physical Activity Level (1 to 5)	2.4 (0.7)	2.6 (0.7)	2.2 (0.7)*
Total Sleep quality (0-21)	4.90 (3.0)	4.38 (2.7)	5.47 (3.1)*
Dimensions of Sleep (0-3)			
Quality	0.95 (0.7)	0.85 (0.7)	1.07 (0.7)*
Latency	0.73 (0.8)	0.62 (0.8)	0.85 (0.9)*
Duration	0.62 (0.8)	0.55 (0.8)	0.71 (0.8)*
Efficiency	0.34 (0.8)	0.33 (0.7)	0.35 (0.8)
Disturbances	1.09 (0.6)	1.02 (0.5)	1.17 (0.6)*
Medication use	0.31 (0.8)	0.28 (0.7)	0.35 (0.8)*
Daytime dysfunction	0.85 (0.8)	0.73 (0.8)	0.98 (0.8)*
Poor sleepers (%)	33.2	26.9	40.3**
Quality of Life (0-100)			
Physical well-being	46.0 (11.9)	49.5 (12.1)	42.2 (10.4)*
Psychosocial well-being	49.6 (12.0)	51.0 (12.3)	48.0 (11.5)*
Mood and emotional state	47.5 (12.8)	49.8 (13.0)	44.9 (12.0)*
Self-perception	47.8 (9.5)	49.3 (9.6)	46.1 (9.1)*
Autonomy	46.7 (11.6)	48.0 (11.9)	45.1 (11.1)*
Parent relationship and family life	48.8 (12.0)	49.1 (12.0)	48.4 (11.9)*
Economic resources	51.0 (9.6)	50.7 (9.7)	51.3 (9.4)*
Friends and social support	52.9 (12.6)	52.7 (12.9)	53.0 (12.3)
School environment	49.0 (10.8)	49.0 (11.2)	48.9 (10.4)
Social acceptance	46.8 (11.6)	46.6 (11.8)	46.9 (11.4)

Both boys and girls who were inactive reported the lowest markers of sleep quality and quality of life, with the highest percentage of poor sleepers observed among this group (see Tables 2, 3).

**Table 2:** Comparison of sleep quality and quality of life according to the performance level in boys Values ​​expressed as mean (standard deviation) or percentage (%). For quantitative variables, the General Linear Model adjusted for age, BMI, municipality size, economic level was used. Post hoc pairwise comparisons were performed with Bonferroni: * p < 0.05 vs. inactive; + p < 0.05 vs. non-competitive; ^ p < 0.05 vs. local football; # p < 0.05 vs. regional football. For qualitative variables, differences were examined using the Chi-square test.

	Inactive (n = 288)	Non-competitive (n = 1394)	Local football (n = 1028)	Regional football (n = 143)	National football (n = 148)	p value
Physical activity/training						
Years of practice		4.1 (3.3)	7.6 (3.1)+	8.9 (2.7)+^	9.1 (2.9)+^#	0.000
Hours/week of practice		4.0 (2.8)	5.8 (3.4)+	6.7 (2.5)+^	7.7 (4.6)+^	0.000
Days/week of practice		3.2 (1.5)	3.6 (1.2)+	3.9 (0.9)+	4.2 (0.9)+^	0.000
Physical Activity Level (1 to 5)		2.5 (0.6)	2.8 (0.6)+	2.7 (0.5)+	2.8 (0.5)+	0.000
Total Sleep quality (0-21)	5.36 (3.2)	4.59 (2.7)*	4.08 (2.5)*+	3.43 (2.2)*+^	3.54 (2.2)*+	0.000
Dimensions of Sleep (0-3)						
Quality	1.10 (0.8)	0.85 (0.7)*	0.79 (0.7)*	0.72 (0.6)*	0.78 (0.6)*	0.000
Latency	0.74 (0.9)	0.67 (0.8)	0.58 (0.7)*	0.45 (0.7)*+	0.46 (0.7)*+	0,000
Duration	0.80 (0.8)	0.62 (0.8)*	0.45 (0.7)*+	0.37 (0.7)*+	0.32 (0.6)*+	0.000
Efficiency	0.36 (0.8)	0.37 (0.8)	0.30 (0.7)	0.20 (0.6)	0.30 (0.7)	0.032
Disturbances	1.06 (0.6)	1.03 (0.5)	1.04 (0.5)	0.87 (0.5)*+^	0.87 (0.5)*+^	0.000
Medication Use	0.28 (0.8)	0.27 (0.7)	0.29 (0.7)	0.24 (0.7)	0.31 (0.7)	0.821
Daytime Dysfunction	1.01 (0.9)	0.79 (0.8)*	0.63 (0.7)*+	0.57 (0.7)*+	0.50 (0.7)*+	0.000
Poor sleepers (%)	39.9	33.1	24.6	15.9	16.8	0.000
Quality of life (0 to 100)						
Physical well-being	37.3 (10.1)	47.9 (11.1)*	53.5 (11.4)*+	54.6 (10.5)*+	55.6 (10.4)*+	0.000
Psychosocial well-being	46.0 (13.9)	50.1 (12.3)*	52.9 (11.6)*+	53.0 (11.1)*+	53.8 (10.9)*+	0.000
Mood and emotional state	47.3 (13.3)	48.7 (12.7)	51.3 (13.1)*+	52.1 (12.2)*+	52.5 (13.3)*+	0.000
Self-perception	48.2 (10.7)	49.1 (9.6)	49.8 (9.5)	49.9 (9.5)	49.9 (9.0)	0.149
Autonomy	44.1 (12.9)	47.3 (11.6)*	49.7 (11.6)*+	49.1 (12.2)*	49.7 (10.4)*	0.000
Parent relationship and family life	45.9 (12.9)	48,1 (12.1)	50.6 (11.7)*+	51.3 (11.1)*+	52.5 (10.6)*+	0.000
Economic resources	48.3 (10.6)	50.1 (9.8)	51.6 (9.3)*+	52.6 (9.4)*+	52.6 (9.0)*+	0.000
Friends and social support	47.9 (14.1)	51.9 (12.6)*	54.7 (12.5)*+	54.7 (13.5)*	53.9 (11.9)*	0.000
School environment	45.8 (11.6)	49.2 (11.4)*	49.9 (10.9)*	48.2 (10.5)	48.2 (9.5)	0.000
Social acceptance	45.3 (11.8)	45.6 (11.8)	47.5 (11.7)*+	49.0 (11.2)*+	49.9 (11.2)*+	0.000

**Table 3:** Comparison of sleep quality and quality of life according to the performance level in girls Values ​​expressed as mean (standard deviation) or percentage (%). For quantitative variables, the General Linear Model adjusted for age, BMI, municipality size, economic level was used. Post hoc pairwise comparisons were performed with Bonferroni: * p < 0.05 vs. inactive; + p < 0.05 vs. non-competitive; ^ p < 0.05 vs. local football; # p < 0.05 vs. regional football. For qualitative variables, differences were examined using the Chi-square test.

	Inactive (n = 743)	Non-competitive (n = 1855)	Local football (n = 52)	Regional football (n = 22)	National football (n = 19)	p value
Physical activity/training						
Years of practice		3.7 (3.1)	4.2 (2.9)	7.3 (3.4)+^	7.6 (3.3) +^	0.000
Hours/week of practice		3.2 (2.4)	4.0 (1.7)	5.7 (2.2)+^	8.2 (7.2)+^#	0.000
Days/week of practice		2.8 (1.5)	3.1 (1.2)	3.6 (0.7)+	3.6 (0.7)+	0.001
Physical Activity Level (1 to 5)	1.6 (0.5)	2.4 (0.6)*	2.4 (0.7)*	2.6 (0.5)*	2.7 (0.8)*	0.000
Total Sleep quality (0-21)	5.78 (3.1)	5.41 (3.1)	4.10 (2.5)*+	5.00 (3.4)	4.05 (2.3)	0.000
Dimensions of Sleep (0-3)						
Quality	1.12 (0.8)	1.05 (0.7)	1.04 (0.7)	1.05 (0.6)	0.95 (0.7)	0.258
Latency	0.84 (0.9)	0.87 (0.9)	0.42 (0.7)*+	0.91 (1.0)	0.63 (0.8)	0.006
Duration	0.83 (0.9)	0.66 (0.8)*	0.67 (0.7)	0.59 (0.7)	0.63 (0.8)	0.000
Efficiency	0.37 (0.8)	0.35 (0.8)	0.19 (0.6)	0.23 (0.4)	0.11 (0.3)	0.257
Disturbances	1.15 (0.5)	1.18 (0.6)	0.92 (0.7)*+	1.18 (0.5)	1.05 (0.5)	0.015
Medication Use	0.34 (0.8)	0.36 (0.8)	0.15 (0.5)	0.32 (0.9)	0.16 (0.5)	0.335
Daytime Dysfunction	1.13 (0.9)	0.94 (0.8)*	0.69 (0.8)*	0.73 (0.8)	0.53 (0.6)*	0.000
Poor sleepers (%)	46.4	41.1	26.4	36.4	21.1	0.000
Quality of life (0 to 100)						
Physical well-being	35.6 (8.0)	44.5 (10.1)*	46.0 (11.1)*	53.6 (9.6)*+^	55.2 (8.9)*+^	0.000
Psychosocial well-being	45.5 (11.7)	49.1 (11.2)*	45.1 (11.2)	49.6 (11.6)	51.9 (10.2)	0.000
Mood and emotional state	43.0 (12.1)	45.6 (11.8)*	43.9 (11.7)	47.3 (12.6)	49.3 (12.5)	0.000
Self-perception	45.3 (9.2)	46.4 (9.1)*	45.1 (7.9)	47.0 (10.3)	51.1 (8.7)*	0.004
Autonomy	43.5 (11.8)	45.7 (10.8)*	45.0 (10.7)	49.0 (9.1)	50.1 (11.8)	0.000
Parent relationship and family life	47.1 (12.1)	49.0 (11.7)*	44.0 (12.8)+	49.8 (9.6)	51.0 (9.4)	0.001
Economic resources	50.2 (9.7)	51.8 (9.3)*	48.4 (10.6)	55.3 (7.6)^	52.2 (7.8)	0.000
Friends and social support	51.2 (12.5)	53.8 (12.0)*	48.4 (13.8)+	55.2 (10.2)	58.3 (9.7)^	0.000
School environment	47.1 (10.6)	49.7 (10.2)*	45.2 (10.8)	45.5 (6.4)	53.1 (12.0)^	0.000
Social acceptance	46.9 (11.5)	47.0 (11.4)	41.3 (11.3)*+	50.4 (11.0)^	48.9 (11.8)	0.001

**Figure 1:**
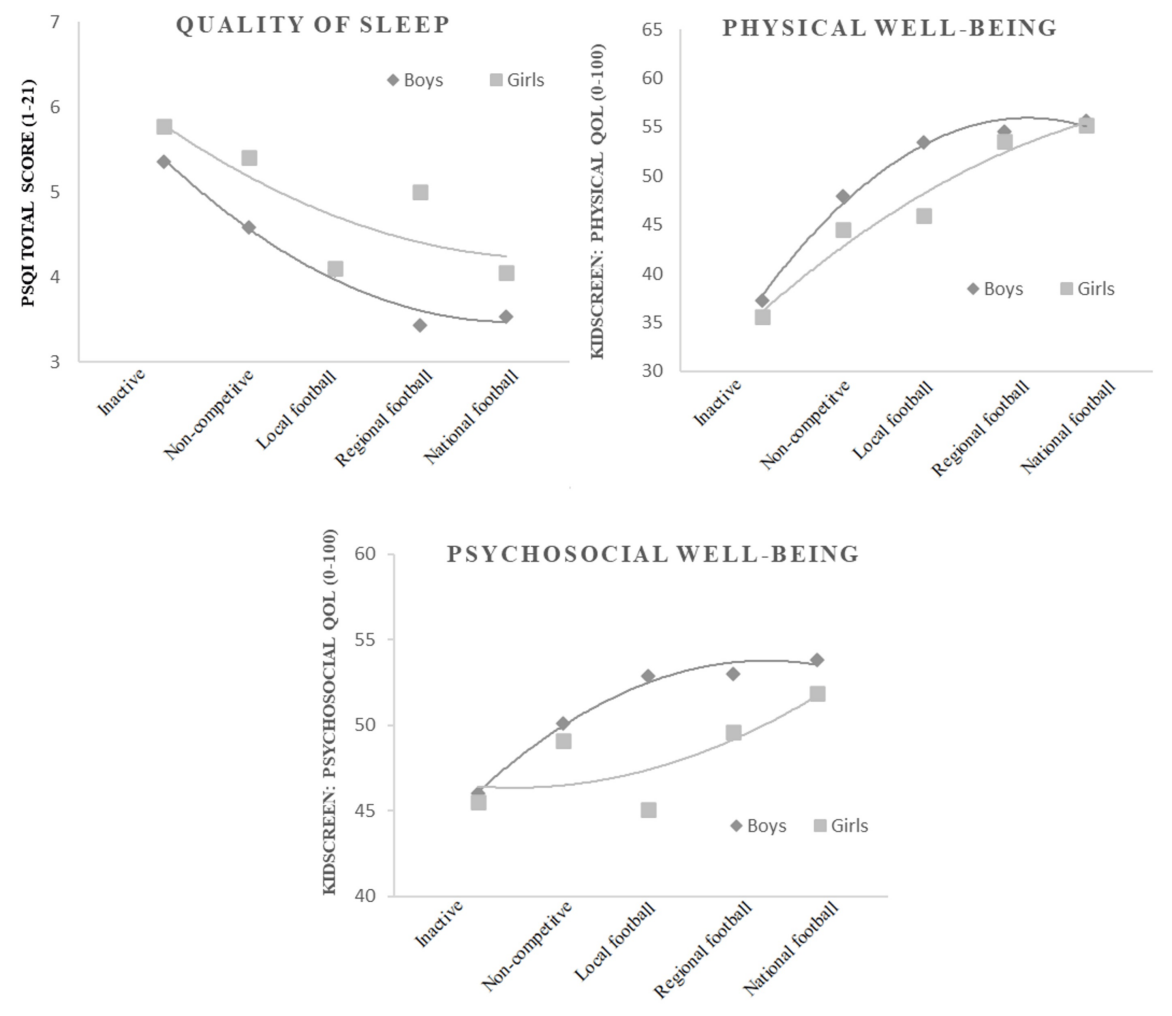
Relationship of quality of sleep, physical and psychosocial well-being with performance level In the Pittsburgh Sleep Quality Index (PSQI) higher scores reflect poorer sleep quality; in the Health-Related Quality of Life Questionnaire for Children and Adolescents (KIDSCREEN), lower scores reflect poorer quality of life.

Among boys, participation in competitive or non-competitive sports generally improves sleep quality. However, soccer players, regardless of their level of competition, reported the best sleep outcomes. Those competing at regional or national levels exhibited the fewest sleep disturbances and the lowest percentage of poor sleepers. Overall, higher levels of competition are associated with better sleep quality, with regional-level soccer players showing the most favorable results (Table 2, Figure 1). In terms of quality of life, soccer players demonstrated the highest levels of physical and psychosocial well-being, with no significant differences between regional and national competitors in sleep and quality of life outcomes (Table 2, Figure 1).

For girls, sports participation, whether competitive or not, generally improves sleep quality. Higher competition levels in soccer were associated with better sleep outcomes (Table 3). Regarding quality of life, there were no substantial differences between girls competing at the local level and those engaging in non-competitive sports. Girls competing at regional and national levels achieved the highest scores in physical well-being and social dimensions (Table 3).

## Discussion

The present study investigated the impact of athletic competition levels on sleep quality and quality of life in adolescent soccer players. Our findings indicate that higher levels of sports competition are associated with significantly improved sleep quality and quality of life among adolescent soccer players, although the extent of these effects varies. Specifically, adolescents who engaged in physical activity, irrespective of their competitive level, demonstrated better sleep quality and quality of life compared to their inactive peers. This observation aligns with existing literature, which suggests that physical activity enhances sleep quality in adolescents and supports sleep restoration [4,25]. Additionally, physical activity has been linked to higher perceived health-related quality of life [9]. Further, a high level of physical activity is consistently associated with better quality of life in adolescents [15].

Our results also reveal that among males, those competing at the regional level exhibited superior sleep quality and quality of life compared to other competitors. Similarly, female soccer players competing at the national level reported the best outcomes in both sleep quality and quality of life. These findings are consistent with previous literature that linked higher levels of physical activity to improved sleep quality [15]. Systematic reviews have shown that higher-level athletes tend to score better on psychological factors such as mental toughness and conscientiousness and experience lower levels of anxiety [26].

The novelty of this study lies in its analysis of how varying levels of competition influence sleep quality and quality of life in adolescent soccer players. Our results suggest that higher levels of competition generally result in better outcomes in both domains, particularly for girls. Previous studies have established that highly competitive sports positively affect sleep quality [15,27,28], and that physical activity can mitigate psychological symptoms through pathways like self-esteem and self-efficacy [29].

However, it is important to note that not all research aligns with our findings. Some studies argue that intense competition may lead to increased stress and anxiety, potentially deteriorating both sleep quality and overall well-being. Previous studies have shown that high levels of competitive stress in youth sports can result in sleep disturbances, anxiety, psychological distress, and burnout, especially due to pre-competition pressure [30].

Given these conflicting results, our findings contribute to an ongoing debate. The variation in outcomes could be attributed to factors such as gender, the nature of the sport, individual coping mechanisms, motivation, self-determination, or the level of support from coaches and peers. Further research is needed to clarify under which conditions competitive sports exert positive or negative effects on adolescent athletes’ well-being. It is important to acknowledge several limitations of this study. First, the use of self-reported data obtained from adolescents, such as surveys, introduces potential biases, including social desirability and recall bias, which may influence the accuracy of the reported sleep quality and quality of life. Furthermore, the cross-sectional design of the study limits our ability to establish causality between competition levels and the observed outcomes. Additionally, the sample size may not be representative of all adolescent athletes, and future studies should consider longitudinal designs and objective measures, such as actigraphy or polysomnography, to provide more robust insights into how competition impacts sleep and well-being over time.

## Conclusions

This study provides valuable insights into how the level of competition in soccer affects sleep quality and quality of life among adolescents. Our findings indicate that participation in soccer, regardless of competitive level, is associated with better sleep quality and quality of life compared to inactivity. Notably, adolescents involved in higher levels of competition, regional or national, demonstrate the most favorable outcomes in both domains. For boys, national and regional-level soccer players exhibited the best sleep quality and the highest quality of life. Similarly, among girls, those competing at regional and national levels reported superior sleep quality and quality of life compared to their counterparts in lower competitive tiers.
